# Protein-Protein Interactions within Late Pre-40S Ribosomes

**DOI:** 10.1371/journal.pone.0016194

**Published:** 2011-01-20

**Authors:** Melody G. Campbell, Katrin Karbstein

**Affiliations:** Department of Cancer Biology, The Scripps Research Institute, Jupiter, Florida, United States of America; University of Queensland, Australia

## Abstract

Ribosome assembly in eukaryotic organisms requires more than 200 assembly factors to facilitate and coordinate rRNA transcription, processing, and folding with the binding of the ribosomal proteins. Many of these assembly factors bind and dissociate at defined times giving rise to discrete assembly intermediates, some of which have been partially characterized with regards to their protein and RNA composition. Here, we have analyzed the protein-protein interactions between the seven assembly factors bound to late cytoplasmic pre-40S ribosomes using recombinant proteins in binding assays. Our data show that these factors form two modules: one comprising Enp1 and the export adaptor Ltv1 near the beak structure, and the second comprising the kinase Rio2, the nuclease Nob1, and a regulatory RNA binding protein Dim2/Pno1 on the front of the head. The GTPase-like Tsr1 and the universally conserved methylase Dim1 are also peripherally connected to this second module. Additionally, in an effort to further define the locations for these essential proteins, we have analyzed the interactions between these assembly factors and six ribosomal proteins: Rps0, Rps3, Rps5, Rps14, Rps15 and Rps29. Together, these results and previous RNA-protein crosslinking data allow us to propose a model for the binding sites of these seven assembly factors. Furthermore, our data show that the essential kinase Rio2 is located at the center of the pre-ribosomal particle and interacts, directly or indirectly, with every other assembly factor, as well as three ribosomal proteins required for cytoplasmic 40S maturation. These data suggest that Rio2 could play a central role in regulating cytoplasmic maturation steps.

## Introduction

Ribosomes, the macromolecular machines that catalyze protein synthesis in all cells, are as complex in their assembly as in their function. In eukaryotes, the two subunits – the small 40S and large 60S – contain 78 ribosomal proteins and four ribosomal RNAs (rRNAs). In addition to these structural components, assembly requires ∼200 conserved, essential accessory factors [1], [2], [3]. These associate transiently with assembling ribosomes in order to facilitate and integrate the processing and folding of rRNA, as well as the binding of ribosomal proteins. However, the precise function of these assembly factors during ribosome assembly remains unknown in many cases.

**Figure 1:**
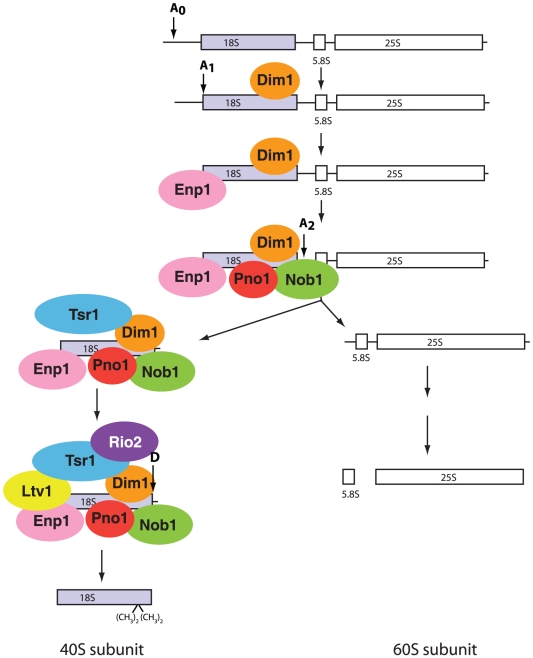
Pre-ribosome processing and assembly scheme. 18S, 5.8S and 25S rRNAs are cotranscribed and the resulting transcript is cleaved in multiple steps to release the mature rRNAs. For simplicity only the 40S processing pathway is shown in any detail, and ribosomal proteins are not depicted. Furthermore, only assembly factors whose interactions are mapped herein are shown. Co-transcriptional cleavage at site A_2_ is preceded by cleavage at sites A_0_ and A_1_. The order of assembly is based on the observation that depletion of Dim1 results in loss of cleavage at site A_1_, and A_2_ [11], while loss of Enp1 and Dim2/Pno1 lead to inhibition of cleavage at sites A_2_ [12], [13]. Loss of all other assembly factors inhibits cleavage at site D [14], [18], [43], [44]. Furthermore, TAP-affinity purification shows that Dim1 and Enp1 purify the largest amount of (early) accessory factors, followed by Tsr1. The pulldowns resulting from TAP-tags on Rio2, Ltv1 and Nob1 are identical ([15], [16] and data not shown). TAP-purification of Dim2/Pno1 associated particles was not possible [16].

A large body of previous work has provided a rough outline of the pathways for 40S and 60S assembly (Figure 1). Three of the four rRNAs are transcribed as a single transcript, which is co-transcriptionally cleaved at site A_2_ [4] to separate the rRNAs destined for the small and large ribosomal subunit. This cleavage step and the preceding cleavages at sites A_0_ and A_1_ are facilitated by binding of several large subcomplexes, including the UtpA, UtpB, UtpC and Imp3/Imp4/Mpp10 complexes, as well as additional proteins such as the late-acting methylase Dim1, the RNA binding protein Dim2/Pno1, and others [5], [6], [7], [8], [9], [10], [11], [12]. All of these form the small subunit (SSU) processosome. At some point prior to cleavage at site A_2_ the assembly factor Enp1 binds, followed by the GTPase-like protein Tsr1. Next, the export adaptor Ltv1, the D-site nuclease Nob1 and the Rio2 kinase associate with pre-ribosomal particles (Figure 1, [13], [14], [15], [16]). These proteins, together with Dim1 and Dim2/Pno1, remain bound to form the 43S pre-ribosome, which is exported into the cytoplasm, where Dim1 methylates two universally conserved adenosines near the 3′-end of 18S rRNA and Nob1 cleaves pre-rRNA at site D, the 3′-end of 18S rRNA (Figure 1). While it is known that these assembly factors (with the exception of Ltv1) are essential and their deletion stalls cytoplasmic maturation of 18S rRNA [13], [14], [17], [18], [19], [20], the specific role of these factors in the cytoplasmic cleavage and maturation of pre-ribosomes remains unclear.

Sequence analysis, combined with knowledge about the rRNA processing intermediate that accumulates upon mutation or depletion of a ribosome assembly factor can often provide a starting point for analyzing the role of an enzyme in ribosome assembly (nucleases, methylases, or helicases). However, for the majority of ribosome assembly factors sequence analysis provide few clues, as most only contain protein-protein interaction motifs, or, in a few cases, RNA-binding motifs. Furthermore, it is likely that at least a subset of these factors plays no direct role in ribosome assembly but instead merely aids the process, either by providing a scaffold for the binding of essential enzymes, or, perhaps by allosterically affecting the function of enzymes, or even by providing “docking” sites for regulatory modules.

Elucidating the ribosome binding sites and interacting partners of ribosome assembly factors can serve as an initial clue in defining their specific function. Towards this goal, Baserga and colleagues have recently undertaken a systematic yeast two-hybrid analysis of the UtpA and UtpB complexes to provide a comprehensive map of protein-protein interactions within these complexes [21], [22]. A potentially complementary approach is a crosslinking technique recently developed by Tollervey and colleagues to map the RNA binding sites of the U3 snRNP, the helicase Prp43, and components of the 43S pre-ribosome [23], [24], [25]. While this map of the rRNA binding sites has been tremendously helpful, especially since the late 43S pre-ribosomal particle is expected to substantially resemble the mature 40S particle, for which good structural information is available [26], [27], it provides little information about interactions between less characterized assembly factors and key factors such as the nuclease Nob1 and the kinase Rio2.

Here we have supplemented the crosslinking approach taken by the Tollervey lab and systematically mapped the protein-protein interactions between the seven non-ribosomal proteins bound to pre-43S ribosomes using recombinant proteins in *in vitro* binding assays. We have also determined the interactions between these factors and several ribosomal proteins with known binding sites in order to map the protein-protein networks onto the structure of the ribosome. This strategy has produced a complete map, within the limits of our technique, of protein-protein interactions between the seven ribosome assembly factors present in 43S pre-ribosomes. These data show that Rio2 interacts with a large number of other ribosome assembly factors, including the nuclease Nob1, the methylase Dim1 and the GTPase-like protein Tsr1. Furthermore, Rio2 directly binds to Rps5, Rps14 and Rps15, ribosomal proteins required for 20S cleavage. Given the locations of these ribosomal proteins, these interactions suggest that Rio2 binds at the head of the pre-ribosome on the subunit interface. Furthermore, the interacting partners we have identified are candidate substrates for the kinase activity of Rio2. Finally, Rio2's central position and ability to interact, directly or indirectly, with all other assembly factors suggests that Rio2 could act as a master-regulator of the Nob1-dependent 20S cleavage step.

## Results and Discussion

**Figure 2:**
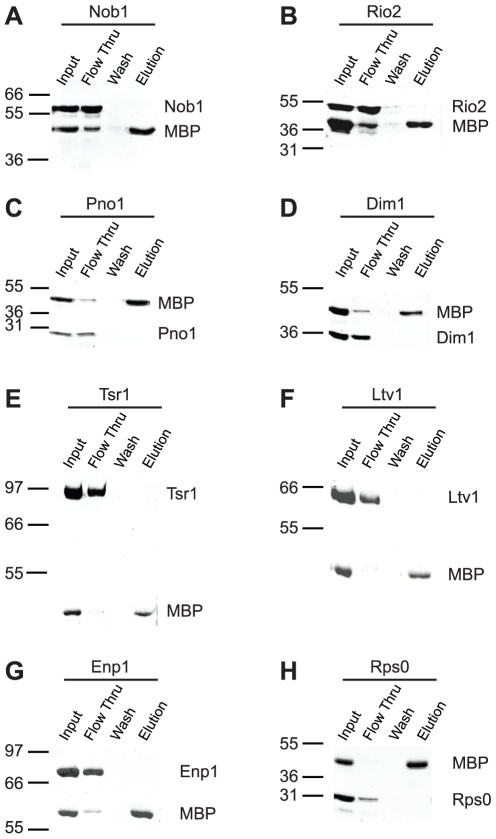
None of the ribosome assembly factors tested interacts with MBP or the resin. Binding assays were performed as described in the Materials and Methods using purified maltose-binding protein (MBP) and untagged assembly factors.

After the early co-transcriptional rRNA cleavages and separation of the rRNAs destined for the large and small subunit, the 40S precursor is exported into the cytoplasm, where the final cleavage to produce the mature 3′-end occurs. This 40S precursor, often referred to as the 43S pre-ribosome, contains most ribosomal proteins present in the mature particle [28], as well as seven assembly factors: Nob1, Rio2, Dim1, Dim2/Pno1, Tsr1, Enp1 and Ltv1 [15], [16]. To determine the protein-protein interactions within these late pre-40S ribosomes, and to provide constraints for their location, we cloned these ribosome assembly factors as well as the ribosomal proteins Rps0, Rps3, Rps5, Rps14, Rps15 and Rps29 from the yeast *S. cerevisiae* and overexpressed them as MBP-fusion proteins in *E.coli*. All ribosomal proteins tested have homologs in bacteria for which their locations are known, and we have used them to roughly position the assembly factors within the pre-ribosomal particles. In this strategy, Rps3 binds at the back of the beak, Rps15 and Rps29 bind on the head towards the beak, Rps5 and Rps14 bind at the platform, and Rps0 at the back of the platform. The tag was removed for all ribosome assembly factors, and proteins were purified over three columns. *In vitro* pulldowns were carried out by incubating MBP-tagged bait protein with untagged prey and amylose beads. After unbound protein was washed out, beads were extensively washed and bound protein was eluted with maltose for analysis by SDS-PAGE. To ensure that the prey proteins did not interact non-specifically with the MBP-tag or amylose resin, control experiments were carried out using each of the untagged proteins and purified MBP (Figure 2). The observation that each untagged protein does *not* bind to at least a few (MBP-)tagged proteins (red in Table S1) further supports the notion that all interactions are specific. All interactions herein (except for those involving ribosomal proteins) were tested both ways, with each protein serving alternatively as bait or as prey.

### Interactions of the nuclease Nob1

**Figure 3:**
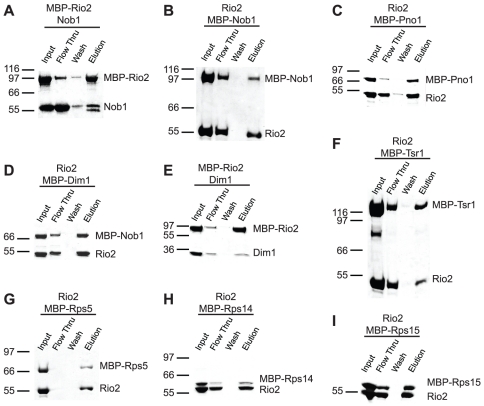
Interactions of Rio2 with ribosome assembly factors and select ribosomal proteins. (A) Nob1 binds (MBP-)Rio2. The doublet in Nob1 arises from proteolytic cleavage of Nob1 near amino acid 454 (data not shown) as also observed previously [39]. (B) Rio2 binds (MBP-)Nob1, the reverse pulldown of (A). (C) Rio2 binds (MBP-)Pno1. (D) Rio2 binds (MBP-)Dim1. (E) Dim1 binds (MBP-)Rio2, the reverse pulldown of (D). (F) Rio2 binds (MBP-)Tsr1. (G) Rio2 binds (MBP-)Rps5. (H) Rio2 binds (MBP-)Rps14. (I) Rio2 binds (MBP-)Rps15.

We have previously defined the ribosome-binding site of the nuclease Nob1 and have shown that it centers around cleavage site D, at the junction between the head and platform domains [29]. In this position, Nob1 makes interactions with the ribosomal proteins Rps5 and Rps14, both of which are required for the Nob1-dependent cleavage step [30], [31]. In addition, we have recently shown that Nob1 directly interacts with the RNA-binding protein Dim2/Pno1 [32], consistent with a previously observed yeast-two-hybrid interaction [33]. To further define Nob1's interactome, we used *in vitro* pulldowns to systematically test interactions between Nob1 and each of the other ribosome assembly factors present in 43S ribosomes. This analysis revealed that in addition to the factors named above, Nob1 also interacts with (MBP-)Rio2 (Figure 3A). This interaction was confirmed by the reverse pulldown between (MBP-)Nob1 and Rio2 (Figure 3B).

Dim2/PnoI has been named Dim2 because of a suggested direct interaction with Dim1 [12], or as Pno1 (Partner-of-Nob1) because of a two-hybrid interaction with Nob1 [33]. Because Dim2/Pno1 interacts with Nob1 [32] but not Dim1, Pno1 may be the more appropriate name and we have chosen this designation in our Figures. The observed *in vivo* interaction between Dim1 and Dim2/Pno1 might represent an indirect interaction within pre-ribosomal particles.

### Interactions of the kinase Rio2

We next examined the interactions of the protein kinase Rio2, one of two kinases required for the final cytoplasmic step in 40S assembly. While Rio2 is essential, its role in ribosome assembly, as well as its substrate, remains unclear [17], [18]. Any substrate for the kinase activity of Rio2 needs to first bind Rio2. Thus, to identify a list of Rio2 substrate candidates, we tested all 43S pre-ribosome assembly factors for interactions with Rio2. Figure 3 shows that in addition to the interaction with Nob1, Rio2 binds directly to Dim1, (MBP-)Dim2/Pno1, and (MBP-)Tsr1, as well as the ribosomal proteins Rps5, Rps14 and Rps15.

In contrast to the interactions between Rio2 and Nob1, and Rio2 and Dim1, reverse pulldowns do not confirm interaction of Rio2 with Dim2/Pno1 or Tsr1 (Table S1). However, we note that an interaction between archeal Dim2/Pno1 and Rio2 has recently been reported [34]. The results with Tsr1 are more ambiguous, as (MBP-)Rio2 (93 kDa) and Tsr1 (91 kDa) run almost identically on an SDS-PAGE, making it difficult to rule out an interaction. Because the tags cannot be cleaved off the ribosomal proteins, as these are otherwise insoluble due to their small size and strong charge, we cannot test the interactions between Rio2 and the ribosomal proteins by reverse tagging. However, the interaction between Rio2 and Nob1, confirmed by reverse pulldowns, combined with the observation that both interact with the ribosomal proteins Rps5 and Rps14, provides further support for the interaction between Rio2 and these ribosomal proteins.

Inability to observe the reverse interactions could signify a) an artifact, b) lack of activity of the other tagged or untagged protein, or c) a steric block from the tag. Because both tagged and untagged Dim2/Pno1 and Rio2 function in other pulldowns, we can rule out lack of activity of these proteins, but not Tsr1. We have carried out careful controls and show that each one of the proteins does not bind to resin or MBP alone (Figure 2), indicating that the possibility of an artifact is low, but cannot be completely excluded. Nevertheless, we believe it is most likely that the location of the MBP-tag is critical for the ability to observe an interaction between Rio2 and Tsr1 as well as Dim2/Pno1. Indeed, data in the literature indicate that N- and C-terminal tags on Nob1 can lead to rRNA processing and growth phenotypes *in vivo* [25].

Ribosomal proteins are generally small, charged and often contain highly positively charged extensions for direct interactions with negatively charged RNA. This opens the possibility for non-specific, charge-mediated interactions with negatively charged proteins. While Nob1 contains an overall positive charge (pI = 8.7), it does contain stretches of negative charge near the C-terminus. Rio2 has an overall negative charge (pI = 5), and could therefore interact non-specifically with ribosomal proteins. Nevertheless, we do not believe that non-specific, charge-mediated interactions are responsible for the observed interactions of Rio2 and Nob1 with ribosomal proteins. This is because similar interactions would be expected to occur with Rps29 (pI = 11.1), or, in the case of Nob1, also Rps15. Furthermore, Tsr1 and Dim2/Pno1 also contain stretches of positive charges, yet neither bind any ribosomal proteins.

### Interactions at the beak

**Figure 4:**
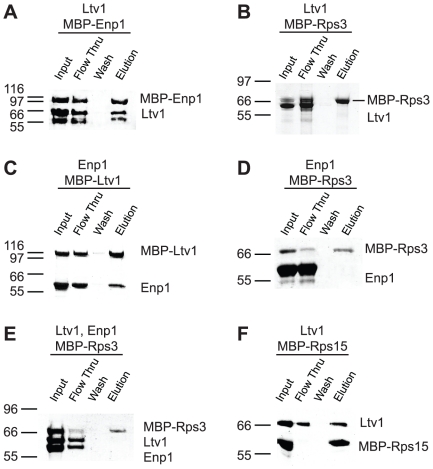
Interactions at the beak. (A) Ltv1 binds (MBP-)Enp1. The observed band below Ltv1 is likely an Ltv1 proteolysis product, observed consistently when different Enp1 and Ltv1 batches are used. (B) Ltv1 does not bind (MBP-)Rps3. (C) Enp1 binds (MBP-)Ltv1, the reverse pulldown of (A). (D) Enp1 does not bind (MBP-)Rps3. (E) A ternary complex between Enp1, Ltv1 and Rps3 is not observed. (F) Ltv1 binds (MBP-)Rps15.

Previous work from the Hurt lab indicated that Enp1 and Ltv1 form a subcomplex containing Rps3, as they co-elute from pre-ribosomal particles at elevated salt concentrations [16]. Furthermore, a two-hybrid interaction between Rps3 and Ltv1 has been reported [35]. To test whether this subcomplex reflected a direct interaction between Enp1 and Ltv1, we performed binding assays as described above. Indeed Ltv1 pulls down (MBP-)Enp1 and Enp1 pulls down (MBP-)Ltv1 (Figure 4A and C, respectively). However, neither protein bound Rps3 in our assay (Figure 4B and D), and we were also unable to assemble a stable ternary complex containing (MBP-)Rps3, Ltv1 and Enp1 (Figure 4E).

It is possible that the MBP-tag on Rps3 inhibits its interaction with Ltv1, Enp1 or both. Alternatively, it is possible that Rps3, Ltv1 or Enp1 must acquire a posttranslational modification that is not present in these recombinantly expressed proteins. Indeed it has been shown that Rps3, Ltv1 and Enp1 are phosphorylated by the kinase Hrr25 [15]. Thus, our data indicate that the interaction between Ltv1, Rps3 and Enp1 might only occur upon phosphorylation of one or more of these proteins, as suggested previously [15].

In addition to the interactions described above, we observe a direct interaction between Ltv1 and the ribosomal protein (MBP-)Rps15 (Figure 4F), which binds on the 60S-facing side of the head, towards the beak. Interestingly, Gleizes and colleagues have recently observed that pre-ribosomal particles purified in the absence of Rps15 lack Rio2 and have reduced amounts of Tsr1 [36], indicating that Rps15 is directly or indirectly required for recruiting these ribosome assembly factors. The data herein suggest that Rps15 might be directly recruiting Rio2, which in turn is bound to Tsr1, rationalizing and extending the previous observations.

Tschochner and colleagues have recently analyzed the composition of subcomplexes of ribosome assembly factors that remain intact after rRNA transcription has been shut off [37]. Their work revealed that Enp1/Ltv1 remained bound to Rio2 and Tsr1. While our data provide direct evidence for the interactions between Ltv1 and Enp1, as well as Tsr1 and Rio2, the only direct link between these protein pairs is Rps15, which binds to both Ltv1 and Rio2. It seems possible that the isolated complexes also contain ribosomal proteins, as these are even more abundant than ribosome assembly factors and also no longer have an rRNA to bind.

**Figure 5:**
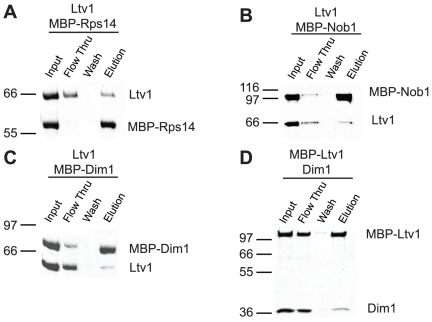
Weak interactions. Ltv1 binds weakly to (MBP-)Rps14 (A), (MBP-)Nob1 (B) and (MBP-)Dim1 (C). (MBP-)Ltv1 also binds Dim1 (D).

We have also observed weak interactions between Ltv1 and (MBP-)Nob1, (MBP-)Rps14 and (MBP-)Dim1, as well as between (MBP-)Ltv1 and Dim1 (light green in Table S1, and Figure 5). While these interactions are substantially less efficient, and thus likely weaker than others presented here, and may thus represent artifacts, they are nevertheless observed reproducibly, and intriguing for three reasons. First, the weak interaction between Ltv1 and Dim1 was observed both with tagged Ltv1 as well as tagged Dim1 (and untagged Dim1, and Ltv1, respectively). Second, all three proteins bind in the same general region of pre-40S ribosomes, as indicated by direct interactions between Nob1 and Rps14 [29], and the observation that the binding site for the bacterial homolog of Dim1 is adjacent to Rps14 [38]. Finally, genetic interactions between Ltv1 and Nob1 have recently been reported [39]. It is unlikely that Ltv1 directly interacts with Nob1, Dim1 and Rps14 in a late 43S particle that resembles mature ribosomes, as their binding sites (when based on the mature structure) are well corroborated and too distant from each other. However, it is possible that these interactions occur in an earlier pre-ribosomal particle, in which either Ltv1 is localized differently or the rRNA is folded differently. In such a postulated earlier particle, the binding sites between Nob1/Rps14/Dim1 and Ltv1 would be sufficiently close for an interaction.

### A protein-network within 43S pre-ribosomes

**Figure 6:**
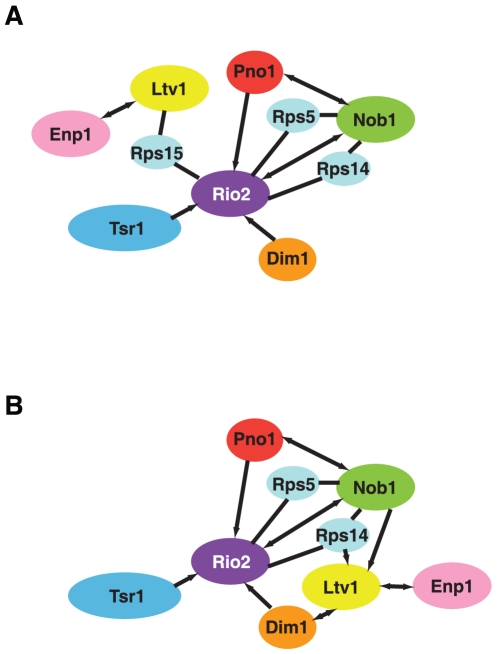
Schematic summary of protein-protein interactions within pre-43S ribosomes. Arrows go from the bait to the prey. Arrows on both sides indicate that reverse pulldowns confirm the interaction. Reverse pulldowns are not possible with ribosomal proteins, as these are not stable untagged. In order to not imply that reverse pulldowns were not successful, these proteins are simply connected by lines. (A) Strong interactions by pulldowns. (B) Weak interactions with Ltv1 are included. Because these cannot be satisfied with the proposed location of Ltv1 near the beak, and the locations for Dim1, Nob1 and Rps14, we have moved Ltv1 in this panel, implying reorganization in this earlier particle.

We have summarized the interactions described herein in a graphic shown in Figure 6A (see also Figure S1). This schematic illustrates the positioning of Rio2 at the heart of the 43S pre-ribosome, where it forms interactions with four of the remaining six assembly factors in 43S preribosomes, as well as with three ribosomal proteins that are required for Nob1-dependent cleavage at site D. Furthermore, the network graphic illustrates the formation of two core modules: the Enp1/Ltv1 module near the beak, and the Rio2/Nob1/Pno1 module. Tsr1 and Dim1 are peripherally connected to this second module. The two modules are connected by the ribosomal protein Rps15, which may be directly involved in recruiting Rio2 to pre-ribosomal complexes [36]. The Rio2/Nob1/Pno1 module is remarkably interconnected, with each of the factors binding to every other one. In addition, both Nob1 and Rio2 bind to the ribosomal proteins Rps5 and Rps14, which are therefore also considered part of this module. Interestingly, both Rps5 and Rps14 appear to bind early to assembling ribosomes, as suggested by the observed defect in 20S accumulation upon depletion of these proteins. It is tempting to speculate that these proteins recruit Dim2/Pno1, which in turn helps recruit Nob1 and, together with Rps15 [36], might recruit Rio2. We also note that the Rio2/Pno1/Nob1 module is conserved in archea, independently corroborating its formation and suggesting its importance in ribosome assembly.

Figure 6B shows an additional scheme that also includes the weak interactions described above. As discussed above, it is unlikely that these can be accommodated in a late particle containing Nob1, Dim1 and Rps14 binding sites near the platform, and Ltv1 near the beak. Instead, we believe they might indicate interactions in an earlier particle. Some of the “strong” interactions may also not be present in that particle, either because proteins are not yet present (perhaps Tsr1 or Rio2), or because the particle is substantially different.

### The binding sites for 43S pre-ribosome assembly factors

**Figure 7:**
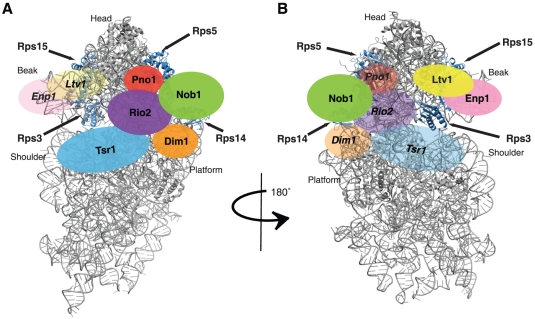
Model for pre-40S subunits with assembly factor binding sites as adapted from [26]. The proposed locations of ribosome assembly factors are shown in the same color scheme as in Figure 6, with Enp1 in pink, Ltv1 in yellow, Rio2 in purple, Dim1 in orange, Nob1 in green, Tsr1 in blue and Pno1 in red. Rps3, Rps5, Rps14 and Rps15 are highlighted in blue. Enp1 and Ltv1 are proposed to bind on the back site. Thus, on the subunit interface they are shown as transparent (A), and on the solvent side as opaque (B). Nob1 is proposed to bind on the side and thus seen in full color on both views. All other proteins are proposed to bind on the subunit interface and are thus shown in full color on the front and in transparent on the solvent side view.

Figure 7 shows the structure of mature 40S ribosomes with the proposed locations of the ribosome assembly factors using the same color scheme for each accessory protein as in Figure 6. This model for the structure of pre-40S ribosomes was built based on the following considerations. First, the binding sites for the ribosomal proteins Rps3, Rps5, Rps14, and Rps15 are known from the recent cryo-EM structure of yeast 40S ribosomes [26]. Furthermore, previous footprinting located the binding site for KsgA, the bacterial Dim1 homolog [38]. This Dim1 binding site is also consistent with recent crosslinking data ([25] and Figure S2). Additionally, we have recently provided a proposal for the Nob1 binding site [29]. Using these fixed locations as a guide, we have placed the remaining proteins onto the structure to satisfy the protein-protein interactions described herein. In addition to the above data, this proposal is consistent with crosslinking data for Enp1 and Rio2 as well as a subset of the observed crosslinks for Tsr1 and Ltv1 ([25] and Figure S2). We also note that in addition to the proposed location for Tsr1, there is at least one alternative additional placement to the right of the decoding site helix that is also consistent with crosslinking data from the Tollervey lab. However, because that location predicts that Tsr1 interacts with Dim1, Rps14, and perhaps also Rps0, Rps5 and Nob1, none of which are observed, we suggest this placement is less likely. Nevertheless, since the (MBP-)tag could interfere with such interactions, the absence of an interaction should not be interpreted too strongly. Finally, we want to stress that the proposed locations, although informative, are only very rough placements and should not be interpreted in molecular terms.

Together, the protein-protein interaction map described here and the crosslinking data from the Tollervey lab suggest that Tsr1, Rio2 and Dim1 bind on the front of the ribosome, as already documented for Dim1 [38]. Further experiments will be necessary to delineate the exact position for these factors, but several intriguing observations can already be made. The proposed location of Tsr1 on the subunit interface is likely to interfere with binding of translation initiation and elongation factors, as well as subunit joining. Thus, a clear prediction would be that Tsr1 should not be found in polysomes, consistent with prior work [40]. Furthermore, the proposed location of Rio2 does not overlap the binding sites for translation initiation factors, consistent with the observation that Rio2 can be found in polysomes [40]. Previous work has already shown that the binding site for Dim1 and IF3 overlap [38].

### The binding partners of the kinase Rio2

Our data indicate that Rio2 is bound on the head, near the mRNA channel, facing the 60S subunit, consistent with crosslinking data ([25], Figure 7). In that position, Rio2 interacts either directly or indirectly with all ribosome assembly factors. Direct interactions are observed with the methylase Dim1, the nuclease Nob1, the GTPase-like protein Tsr1, and the RNA-binding protein Dim2/Pno1. Interactions with Ltv1 and Enp1 are mediated by the ribosomal protein Rps15, which shares interactions with Rio2 and Ltv1. In addition, Rio2 interacts directly with the ribosomal proteins Rps5 and Rps14 (Figures 3 and 6). Deletion of Rps15, as well as small truncations or point mutations in Rps5 and Rps14, all result in inhibition of the Nob1-dependent final cleavage step at the 3′-end of 18S rRNA [30], [31], [41]. Thus, Rio2 is positioned to regulate the Nob1-dependent cleavage, but also, potentially, to be regulated by several of the key proteins in the final step of 40S maturation.

While it is not yet clear whether or not the kinase activity of Rio2 is essential (reviewed in [3]), point mutations in the kinase domain have subtle but distinct effects on ribosome assembly in human cells, providing evidence that phosphorylation by Rio2 at least accelerates processes in late 40S assembly [42]. Nevertheless, substrates of Rio2 have not yet been identified. Given that Nob1, Dim1, Dim2 and Tsr1, as well as the crucial ribosomal proteins Rps5, Rps14 and Rps15, all directly bind to Rio2, these are prime candidates for being substrates of Rio2. Further experiments will be required to test the effect of these proteins on the kinase activity of Rio2.

## Materials and Methods

### Cloning of ribosome assembly factors and ribosomal proteins

The genes for Enp1, Ltv1, Rps3 and Rps29 were PCR-amplified from genomic DNA and cloned between the *SfoI* and *BamHI* sites of pSV272. The genes encoding Dim1, Dim2/Pno1, Tsr1 and Rps15 were PCR-amplified from genomic DNA and cloned between the *SfoI* and *HindIII* sites of pSV272. The gene for Rio2 was PCR-amplified from genomic DNA and cloned between the *SfoI* and *BamHI* sites of pSV272 for (MBP-)Rio2, or between the *NheI* and *BamHI* sites of pET23a for untagged Rio2. Cloning primers are listed in Table 1. Nob1, (MBP-)Rps0, (MBP-)Rps5, and (MBP-)Rps14 were previously cloned and purified as described [29].

**Table 1:** Oligonucleotides Used.

Description	Sequence
5′Dim1SfoI	GATCGAGGCGCCATGGGAAAGGCTG
3′Dim1HindIII	TCAGACAAGCTTTCATGAAAAATGGATACCAAC
5′Enp1SfoI	GATCGAGGCGCCATGGCCAGAGCAT
3′Enp1BamHI	TCAGACGGATCCTCAGACGGATCC
5′Ltv1SfoI	GATCGAGGCGCCATGTCGAAGAAAT
3′Ltv1BamHI	CTACTAGGATCCCTAAAATTTTAAGCTGCTTAGTGTATTG
5′Tsr1SfoI	GATCGAGGCGCCATGGCAGGTCATTC
3′Tsr1HindIII	TCAGACAAGCTTTTACATACCATTCCAAGGTAAC
5′Rio2NheI	GATCGAGCTAGCATGAAATTGGATACTTCTCATAT
5′Rio2SfoI	GATCGAGGCGCCATGAAATTGGATA
3′Rio2BamHi	TCAGACGGATCCCTACTCTAGTATATAGTTTCC
5′Rps3SfoI	GATCGAGCCGCCATGGTCGCTTTAATCTCTAAG
3′Rps3BamHI	CTACTAGGATCCCTAAGCTTCAACTGGTTCAG
5′Rps15SfoI	CCACCAGGCGCCTCTCAAGCTGTTAATGCC
3′Rps15HindIII	CCACCAAAGCTTTTATTTCAATGGGATGAAACG
5′Rps29SfoI	GATCGAGGCGCCATGGCTCACGAAAACGTCTG
3′Rps29BamHI	CTACTAGGATCCTTATCTGAATTTGTTGAAACCAATGTCG

### Overexpression and Purification of Proteins

Unless otherwise noted, proteins were overexpressed in Rosetta cells (Novagen) as follows: LB Miller medium supplemented with 0.05 mM kanamycin and 0.05 mM chloramphenicol was inoculated with overnight cultures and grown at 37°C to an OD_600_ of ∼0.5 before induction with 1 mM IPTG. After 5 h of growth at 30°C the cells were spun down and the pellets resuspended in lysis buffer (according to the Qiagen protein-purification handbook) supplemented with 0.1 mM PMSF and 5 mM benzamidine. Unless otherwise noted, after sonication, the soluble fraction was purified over Ni-NTA resin (Qiagen) in accordance with the manufacturer's handbook. Also, unless otherwise noted, the protein was further purified by ion-exchange chromatography, followed by gel filtration using a Superdex200 column (GE Healthcare), equilibrated with 50 mM HEPES, pH 7.5, 200 mM KCl, 1 mM DTT, and 1 mM TCEP. For long-term storage glycerol was added to 15% and the purified protein was flash frozen in liquid nitrogen and stored at −80°C.

#### Dim1

Dim1 was overexpressed for 12 hours at 17°C. In order to cleave after the His_6_-MBP tag, TEV protease was added to the Ni-eluate containing Dim1 during an overnight dialysis in a buffered solution containing 50 mM K_2_HPO_4_, pH 8.0, 300 mM NaCl, 1 mM DTT and 10% glycerol. Next, the protein was purified over a 20 mL MonoS column in a linear gradient from 150 to 870 mM KCl over 12 column volumes, followed by gel-filtration. Protein concentration was determined by absorption at 280 nm using an extinction coefficient of 21,430 M^−1^cm^−1^. (MBP-)Dim1 was overexpressed and purified as specified above for the cleaved protein, except that following the Ni-NTA column, the protein was dialyzed in 50 mM KHPO4, pH 8.0, 100 mM NaCl, 1 mM DTT, 1 mM TCEP without TEV protease. Protein concentration was determined by absorption at 280 nm using a calculated extinction coefficient of 90,760 M^−1^cm^−1^.

#### Dim2/Pno1

Cells were lysed in 50 mM NaPO_4_, pH 8.0, 150 mM NaCl, and 0.5 mM PMSF and purified via Ni-NTA chromatography using 1 ml HisTrap columns (GE Healthcare). Ni eluate fractions containing Dim2/Pno1 were pooled and dialyzed in the presence of TEV into 50 mM Tris, pH 8.0, 150 mM NaCl and 1 mM DTT. Dialyzed protein was purified over a MonoS column in a 150 mM to 890 mM NaCl salt gradient over 12 column volumes, followed by gel-filtration. Protein concentration was determined by absorption at 280 nm using a calculated extinction coefficient of ε = 11,460 M^−1^cm^−1^. To obtain (MBP-)Dim2, TEV protease was omitted during the dialysis step and protein concentration was determined using a calculated extinction coefficient of ε = 76,180 M^−1^cm^−1^.

#### Enp1

The Ni-eluate containing Enp1 was dialyzed overnight with TEV in a buffered solution containing 30 mM Na_2_HPO_4_, pH 8, 300 mM NaCl and 1 mM DTT. The protein was purified over an 8 mL MonoQ column in a linear gradient from 330 mM to 501 mM KCl over 4 column volumes, followed by gel-filtration. Protein concentration was determined by absorption at 280 nm using an extinction coefficient of 47,330 M^−1^cm^−1^. To purify (MBP-)Enp1 the eluate from the Ni-column was dialyzed overnight against 50 mM K_2_HPO_4_, pH 8.0, 300 mM NaCl and 1 mM DTT. The protein was purified over a MonoQ column in a linear gradient from 100 to 820 mM KCl over 13 column volumes, followed by gel filtration in 300 mM KCl, and 50 mM HEPES, 1 mM DTT and 1 mM TCEP. Protein concentration was determined by absorption at 280 nm using an extinction coefficient of 116,660 M^−1^cm^−1^.

#### Ltv1

Ni-eluate containing Ltv1 was dialyzed overnight with TEV in a buffered solution containing 50 mM Tris-HCl, pH 7.5, 100 mM KCl and 1 mM DTT. The protein was purified over the MonoQ column in a linear gradient from 100 to 640 mM KCl over 12 column volumes, followed by gel-filtration. Protein concentration was determined by absorption at 280 nm using an extinction coefficient of 29,340 M^−1^cm^−1^. (MBP-)Ltv1 was overexpressed and purified as described above for the cleaved protein except the TEV protease was omitted from the overnight dialysis following the Ni-NTA column and a linear gradient of 100 to 1000 mM KCl was run over 20 CV on the MonoQ column. Protein concentration was determined by absorption at 280 nm using a calculated extinction coefficient of 98,670 M^−1^cm^−1^.

#### (MBP-)Nob1

(MBP-)Nob1 was overexpressed and purified as described [29] except the TEV protease was omitted from the overnight dialysis following the Ni-NTA column. Protein concentration was determined using a calculated extinction coefficient of 120,210 M^−1^cm^−1^.

#### Rio2

After sonication, the soluble fraction was purified over a 5 mL Hi-Trap Q column (GE Healthcare) in a linear gradient from 300 to 1000 mM KCl over 20 column volumes. Fractions containing Rio2 were pooled, concentrated, and dialyzed for 3 hours in 50 mM Tris, pH 8, 300 mM KCl, and 2 mM EDTA. The protein was purified over the MonoQ column in a linear gradient from 150 to 870 mM KCl over 13 column volumes, followed by gel-filtration. Protein concentration was determined by absorption at 280 nm using an extinction coefficient of 39,770 M^−1^cm^−1^. (MBP-)Rio2 was purified over a Ni column, then by a 8 mL MonoQ column in a linear gradient from 150 to 870 mM KCl over 13 column volumes, followed by gel-filtration. Protein concentration was determined by absorption at 280 nm using an extinction coefficient of 109,100 M^−1^cm^−1^.

#### Tsr1

Tsr1-containing Ni-eluate was dialyzed overnight with TEV in a buffered solution containing 50 mM Tris, pH 8, 100 mM KCl, and 1 mM DTT. The protein was purified over an 8 mL MonoQ column in a linear gradient from 100 to 640 mM KCl over 13 column volumes, followed by gel-filtration. Protein concentration was determined by absorption at 280 nm using an extinction coefficient of 86,750 M^−1^cm^−1^. For (MBP-)Tsr1 the TEV protease was omitted from the overnight dialysis following the Ni-NTA column, and protein concentration was determined by absorption at 280 nm using a calculated extinction coefficient of 156,080 M^−1^cm^−1^.

#### Rps0

Rps0 was overexpressed and purified as described [29] except TEV protease was added the overnight dialysis following the Ni-NTA column. Protein concentration was determined using a calculated extinction coefficient of 3,644 M^−1^cm^−1^.

#### (MBP-)Rps3

Ni-eluate containing (MBP-)Rps3 was dialyzed overnight in a buffered solution containing 50 mM Tris, pH 7.6, 50 mM KCl, and 1 mM DTT, and then purified over a MonoS column in a gradient from 50 mM to 1 M KCl over 20 column volumes, followed by gel-filtration. Protein concentration was determined by absorption at 280 nm using an extinction coefficient of 79,760 M^−1^cm^−1^.

#### (MBP-)Rps15

Ni-eluate containing (MBP-)Rps15 was dialyzed overnight in a buffered solution containing 50 mM Tris, pH 8, 100 mM KCl, and 1 mM DTT. The protein was purified over an 8 mL MonoQ column in a linear gradient from 100 to 820 mM NaCl over 16 column volumes, followed by gel-filtration. Protein concentration was determined by absorption at 280 nm using an extinction coefficient of 75,290 M^−1^cm^−1^.

#### (MBP-)Rps29

Ni-eluate containing (MBP-)Rps29 was dialyzed overnight in a buffered solution containing 50 mM Tris, pH 8, 150 mM KCl, and 1 mM DTT. The protein was purified over an 8 mL MonoQ column in a linear gradient from 50 to 810 mM KCl over 16 column volumes, followed by gel-filtration. Protein concentration was determined by absorption at 280 nm using an extinction coefficient of 77,810 M^−1^cm^−1^.

### Protein Binding Assays

100 µL solution containing 5 µM untagged protein and 3 µM MBP-tagged protein was made in 50 mM HEPES, pH 7.5, 150 mM KCl. After 10 minutes of incubation at room temperature, the solution was added to 25 µL of amylose resin (New England Biolabs) equilibrated in binding buffer and incubated at 4°C on a rotator mixer for 1 hour. The slurry was loaded onto a Bio-Spin disposable chromatography column, washed three times with 100 µL and a final time with 25 µL of binding buffer A. To elute, 25 µL of 75 mM HEPES, pH 7.5, 150 mM NaCl, 20 mM maltose was added to the column and incubated for 5 minutes before collection. Following the initial flow-through, the final wash and the elution, the column was microcentrifuged for 10 s to collect the sample. Samples were analyzed on SDS PAGE gels ranging from 4% to 12% acrylamide and the protein bands visualized with Coomassie Brilliant blue stain (BioRad).

## Supporting Information

Figure S1 **Comparison of protein interaction data herein to previously observed data.** See text for references.
(EPS)

Figure S2 **Comparison of protein-protein interaction data herein to previously observed RNA-protein interaction data from Ref 25.** (A) Mapping of the crosslinking data observed in Ref. 25 onto the tertiary structure of mature ribosomes. Crosslinks with individual assembly factors are shown in the same color scheme as shown below in panel B. In order to filter noise out of the data, only direct crosslinks, which lead to insertions or deletions in the reverse transcription data, are shown. Furthermore, only those crosslinks are shown that fit in either one of the following two categories: (1) crosslinks were observed at least twice, or (2) at least three crosslinks in a 10 nucleotide window were obtained. The left panel shows the subunit interface side, while the right panel shows the solvent side. We placed ribosome assembly factors onto this structure in accordance with the crosslinking data observed herein (see also Figure 7). (B) Figure 6 is reproduced to aid in the comparison between crosslinking data from Ref. 25 and our data herein.
(EPS)

Table S1 **Summary of Protein-Protein Interactions.**
(DOCX)
