# Supplementary material for: Protein-Protein Interactions within Late Pre-40S Ribosomes
Source: PLoS One. 2011 Jan 20;6(1):e16194. doi: 10.1371/journal.pone.0016194 (PMC3024409; doi:10.1371/journal.pone.0016194)
Supplement: Table S1 — Summary of Protein-Protein Interactions. (DOCX) [file pone.0016194.s003.docx]

Table S1: Summary of Protein-Protein Interactions

|  | Nob1 | Rio2 | Pno1 | Dim1 | Tsr1 | Ltv1 | Enp1 | Rps0 |
| --- | --- | --- | --- | --- | --- | --- | --- | --- |
| MBP-Nob1 |  |  | ** |  |  |  |  |  |
| MBP-Rio2 |  |  |  |  |  |  |  |  |
| MBP-Pno1 | ** |  |  |  |  |  |  |  |
| MBP-Dim1 |  |  |  |  |  |  |  |  |
| MBP-Tsr1 |  |  |  |  |  |  |  |  |
| MBP-Ltv1 |  |  |  |  |  |  |  |  |
| MBP-Enp1 |  |  |  |  |  |  |  |  |
| MBP-Rps0 | * |  |  |  |  |  |  |  |
| MBP-Rps3 |  |  |  |  |  |  |  |  |
| MBP-Rps5 | * |  |  |  |  |  |  |  |
| MBP-Rps14 | * |  |  |  |  |  |  |  |
| MBP-Rps15 |  |  |  |  |  |  |  |  |
| MBP-Rps29 |  |  |  |  |  |  |  |  |

All interactions were tested at least twice via *in vitro* binding assays as described in the Materials and Methods. Green signifies an interaction. Exemplary SDS-Page gels for all interactions are shown in Figures 3 and 4, as well as elsewhere (*[29] and **[Woolls, in the press #2824]). Light green shows weak interactions (Figure 5). Red shows non-detectable interactions. Grey are interactions that have not been tested.
